# Characterization, dissolution and solubility of synthetic cadmium hydroxylapatite [Cd_5_(PO_4_)_3_OH] at 25–45°C

**DOI:** 10.1186/s12932-015-0025-1

**Published:** 2015-07-18

**Authors:** Yinian Zhu, Zongqiang Zhu, Xin Zhao, Yanpeng Liang, Liuqin Dai, Yanhua Huang

**Affiliations:** College of Environmental Science and Engineering, Guilin University of Technology, Guilin, 541004 People’s Republic of China; College of Light Industry and Food Engineering, Guangxi University, Nanning, 530004 People’s Republic of China

**Keywords:** Cadmium hydroxylapatite, Dissolution mechanism, Solubility product, Gibbs free energy of forming

## Abstract

**Background:**

The substitution of Ca^2+^ in Ca-hydroxylapatite by toxic Cd^2+^ can cause the forming of Cd-hydroxylapatite and is a significant issue in a great variety of research areas, which hence needs an understanding of the essential physicochemical characteristics. Unfortunately, the solubility product and thermodynamic data for Cd-hydroxylapatite in water under a variety of conditions now are lacking. Little information has been reported by previous researchers. Additionally, the dissolution mechanism of Cd-hydroxylapatite has never been studied.

**Results:**

Dissolution of the synthetic cadmium hydroxylapatite [Cd-HAP, Cd_5_(PO_4_)_3_OH] in HNO_3_ solution (pH = 2), ultrapure water (pH = 5.6) and NaOH solution (pH = 9) was experimentally studied at 25, 35 and 45°C. Characterization by XRD, FT-IR and FE-SEM proved that Cd-HAP solids showed no recognizable change during dissolution. For the Cd-HAP dissolution in aqueous acidic media at initial pH 2 and 25°C, the solution cadmium and phosphate concentrations increased rapidly and reached the peak values after 20–30 days and 10 days reaction, respectively. Thereafter, the Cd-HAP dissolution rate decreased slowly, whereas the solution Cd/P molar ratio increased constantly from 1.65–1.69 to 6.61–6.76. The mean *K*_sp_ values for Cd_5_(PO_4_)_3_OH were determined to be 10^−64.62^ (10^−64.53^–10^−64.71^) at 25°C, 10^−65.58^ (10^−65.31^–10^−65.80^) at 35°C and 10^−66.57^ (10^−66.24^–10^−66.90^) at 45°C. Based on the obtained solubility data from the dissolution at initial pH 2 and 25°C, the Gibbs free energy of Cd_5_(PO_4_)_3_OH forming $\Delta G_{f}^{o}$ was determined to be −3,970.47 kJ/mol (−3,969.92 to −3,970.96 kJ/mol). Thermodynamic parameters, Δ*G*^0^, Δ*H*^0^, Δ*S*^0^, and $\Delta C_{\text{p}}^{ 0}$ for the dissolution process of Cd-HAP in aqueous acidic media at initial pH 2 and 25°C were calculated 368,710.12 J/K mol, −158,809.54 J/mol, −1,770.20 and −869.53 J/K mol, respectively.

**Conclusions:**

Based on the experimental results of the present work and some previous researches, the cadmium hydroxylapatite (Cd-HAP) dissolution in aqueous media is considered to have the following coincident processes: the stoichiometric dissolution coupled with protonation and complexation reactions, the non-stoichiometric dissolution with Cd^2+^ release and PO_4_^3−^ sorption and the sorption of Cd^2+^ and PO_4_^3−^ species from solution backwards onto Cd-HAP surface. The obtained solubility products (*K*_sp_) 10^−64.62^ (10^−64.53^–10^−64.71^) for Cd-HAP was approximately 7.62–5.62 log units lower than 10^−57^–10^−59^ for calcium hydroxylapatite (Ca-HAP).

**Graphical abstract:**
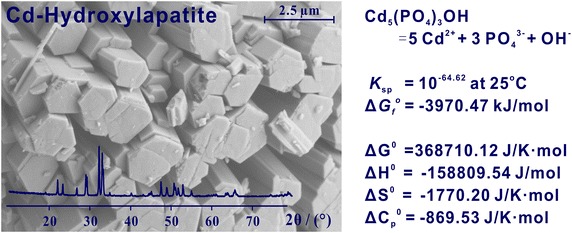
Dissolution of cadmium hydroxylapatite [Cd_5_(PO_4_)_3_OH].

## Background

The apatite group minerals are famous for their compositional changes owing to various substitutions and form an enormous family of isomorphic compounds [1], which play an important role in numerous medical, industrial and environmental processes [2, 3]. Calcium hydroxylapatite [Ca-HAP, Ca_5_(PO_4_)_3_(OH)] is a principal component of teeth and bones of vertebral animals. It is isostructural with calcium hydroxylapatite, and Cd^2+^ has the probability of replacement for Ca^2+^ in hard tissue [4]. Apatites as the raw material for the phosphatic fertilizer production have usually trace amount of many elements, among which Cd is particularly harmful and may be found in natural waters. Cadmium can be concentrated in the animal bone tissues through the food chain, which may cause bone troubles or damage such as osteomalacia and osteoporosis [5]. Ca^2+^ (0.100 nm radius) sites of Ca-HAP can be substituted by Cd^2+^ with 0.095 nm radius that may result in a contraction of the apatitic structure [6]. When toxic Cd^2+^ ions are taken into an organism, they could be possibly concentrated in body and form Ca–Cd hydroxylapatite solid solution [5]. Because of its ion exchangeability to hold many noxious trace elements, Ca-HAP has been used to remove harmful heavy metal ions from industrial wastewaters and to remedy metal-contaminated soils [7–10].

It possesses a large capacity for replacements of calcium and phosphate ions by other ions owing to its chemical composition and crystal structure [11], among which the replacement of Ca^2+^ by poison Cd^2+^ can cause the forming of Cd-HAP. Consequently, the substitution of Ca^2+^ in Ca-HAP by toxic Cd^2+^ is a significant issue in a great variety of research areas [5, 12], which hence needs an understanding of the essential physicochemical characteristics, particularly the dissolution mechanism and solubility under different conditions. Although a great number of experimental studies on the dissolution mechanisms and kinetics of natural and synthetic apatite samples in pure water, acidic and alkali solutions have already been carried out [8, 9, 13–16], much of them has been only focused on calcium hydroxylapatite and fluorapatite. Unfortunately, dissolution mechanism, solubility product and thermodynamic data of Cd-HAP in water under a variety of conditions now are lacking, although its dissolution and following release of the component from the solid to the aqueous solution play a significant role in the cycling of the relevant elements.

Only a few experimental works have been made on the dissolution and stability of Cd-HAP, for which little information has been reported by previous researchers. Solubility studies of cadmium hydroxylapatite (Cd-HAP) were carried out in an aqueous solution of 0.165 M sodium nitrate [17], and the change of solubility products (*K*_sp_) of Cd-HAP with temperatures from 37 to 52°C at a pH of 5.0 was examined [17]. However, there’s still mistake and confusion about their results, e.g., the solubility products (*K*_sp_) for CdHPO and CaHPO were used to calculate the thermodynamic properties, Δ*G*^0^, Δ*H*^0^, Δ*S*^0^, and $\Delta C_{\text{p}}^{0}$ for the dissolution processes of Cd-HAP and Ca-HAP at pH 5.0, respectively [17]. Therefore, there are lacking thermodynamic data that can be used to evaluate the environmental risk of cadmium caused by cadmium hydroxylapatite. Therefore, Cd-HAP was firstly synthesized by precipitation technique and examined by XRD, FT-IR and FE-SEM in this work, and then its dissolution rate and mechanism were investigated at initial pH 2–9 and 25–45°C. Furthermore, the solution concentrations were used to calculate the solubility product and free energy of forming of Cd-HAP. As a comparison, similar experimental work was also done for pure synthetic Ca-HAP.

## Experimental methods

### Solid preparation and characterization

#### Solid preparation

The pure Cd-HAP solid sample was prepared by precipitation according to the following reaction: 5Cd^2+^+3PO_4_^3−^ + OH^−^ = Cd_5_(PO_4_)_3_OH [18]. The synthetic procedure for the Cd-HAP solid by precipitation was based on 50 mL of 0.02 mol/L cadmium solution was firstly prepared by dissolving cadmium acetate dihydrate [Cd(CH_3_COO)_2_·2H_2_O, analytical grade] in pure water. The cadmium solution was then added into 50 mL of 0.22 mol/L ammonium acetate [CH_3_COONH_4_] buffer solution in a 250 mL polypropylene vessel. Thereafter, 100 mL 0.006 mol/L NH_4_H_2_PO_4_ solution was quickly added into the vessel with stirring to form white suspension, with the result that the mixed solution had a Cd/P atomic ratio of 1.67. The resulting mixed solution was adjusted to pH of 7.5 by adding NH_4_OH solution and stirred for 10 min at 23 ± 1°C, and aged at 100°C for 2 days. Subsequently, the white precipitate was rinsed carefully with pure water and then dried in an electric oven at 70°C for 16 h.

#### Characterization

10 mg of the prepared Cd-HAP solid was digested in 20 mL of 1 M HNO_3_ solution and diluted to 100 mL with pure water. The Cd and P contents were then examined by using an inductively coupled plasma—optical emission spectrometer (ICP-OES, Perkin-Elmer Optima 7000DV). The X-ray diffraction (XRD) patterns of the synthetic Cd-HAP powder was recorded by an X’Pert PRO diffractometer with Cu Kα radiation (40 kV and 40 mA), and then identified crystallographically via comparing the obtained XRD pattern with the ICDD reference code 00-014-0302 for cadmium hydroxylapatite. The morphologies of the synthetic Cd-HAP powders were examined through a field-emission scanning electron microscope (Hitachi FE-SEM S-4800). The Fourier transform infrared spectra of Cd-HAP were obtained by an FT-IR spectrophotometer (Nicolet Nexus 470) with KBr pellets within the range of 4,000–400 cm^−1^.

### Dissolution experiments

2.0 g of the prepared Cd-HAP was put into each of 100 mL polypropylene bottles, and then 100 mL of HNO_3_ solution (pH = 2), ultrapure water (pH = 5.6) or NaOH solution (pH = 9) was added. After that, all the bottles were capped and soaked in three constant temperature water baths (25, 35 or 45°C). From each bottle, the reaction solutions (5 mL) were sampled at 22 time intervals (1, 3, 6, 12 h, 1, 2, 2, 5, 10, 15, 20, 30, 45, 60, 75, 90, 120, 150, 180, 210, 240, 300 days), filtered through 0.22 µm pore filters and stabilized in 25 mL volumetric flask by using 0.2% HNO_3_. An equivalent volume of pure water was added after each sampling. The dilution effects of the acidic and basic solutions throughout the experiments were considered in the calculation by using the program PHREEQC [19]. The solution Cd and P concentrations were measured using an ICP-OES instrument. After 7,200 h dissolution, the Cd-HAP solids were sampled from the bottles, rinsed, dried and investigated by XRD, FE-SEM and FT-IR instruments. All further tests were done using nitrate media to minimize the precipitation of impurity phases and to prevent the complexation of the metal ions at higher concentration.

### Thermodynamic calculations

The aqueous activities of Cd^2+^(aq), PO_4_^3−^(aq), and OH^−^(aq) were first determined using PHREEQC Version 3 with the minteq.v4.dat database [19], and then the ion activity products (IAPs) for Cd_5_(PO_4_)_3_OH were calculated according to the mass-action expressions. The minteq.v4.dat database contains thermodynamic data for the aqueous species and gas and mineral phases that are derived from the database files of MINTEQA2. The aqueous species considered for the total cadmium calculation included Cd^2+^, CdOH^+^, Cd(OH)_2_^0^, Cd(OH)_3_^−^, Cd(OH)_4_^2−^ and Cd_2_OH^3+^. For the total phosphate, the species considered were PO_4_^3−^, HPO_4_^2−^, H_2_PO_4_^−^ and H_3_PO_4_^0^. The saturation indexes were calculated for Cd_3_(PO_4_)_2_, Cd(OH)_2_(am), Cd(OH)_2_ and monteponite (CdO). The ionic strength of the solutions during the experiments was always less than 0.023 mol/L, so the Debye–Hückel equation or the extended Debye–Hückel equations were used in the simulation.

## Results and discussion

### Solid characterization

The chemical component of the prepared Cd-HAP is related to the Cd/P molar ratio of the mixing solutions. The Cd_5_(PO_4_)_3_OH precipitation should be carried out by mixing the cadmium solution with the phosphate solution very slowly. The prepared crystal in this work was confirmed to be the intended composition of Cd_5_(PO_4_)_3_OH with the atomic Cd/P ratio of 1.67.

**Figure 1:**
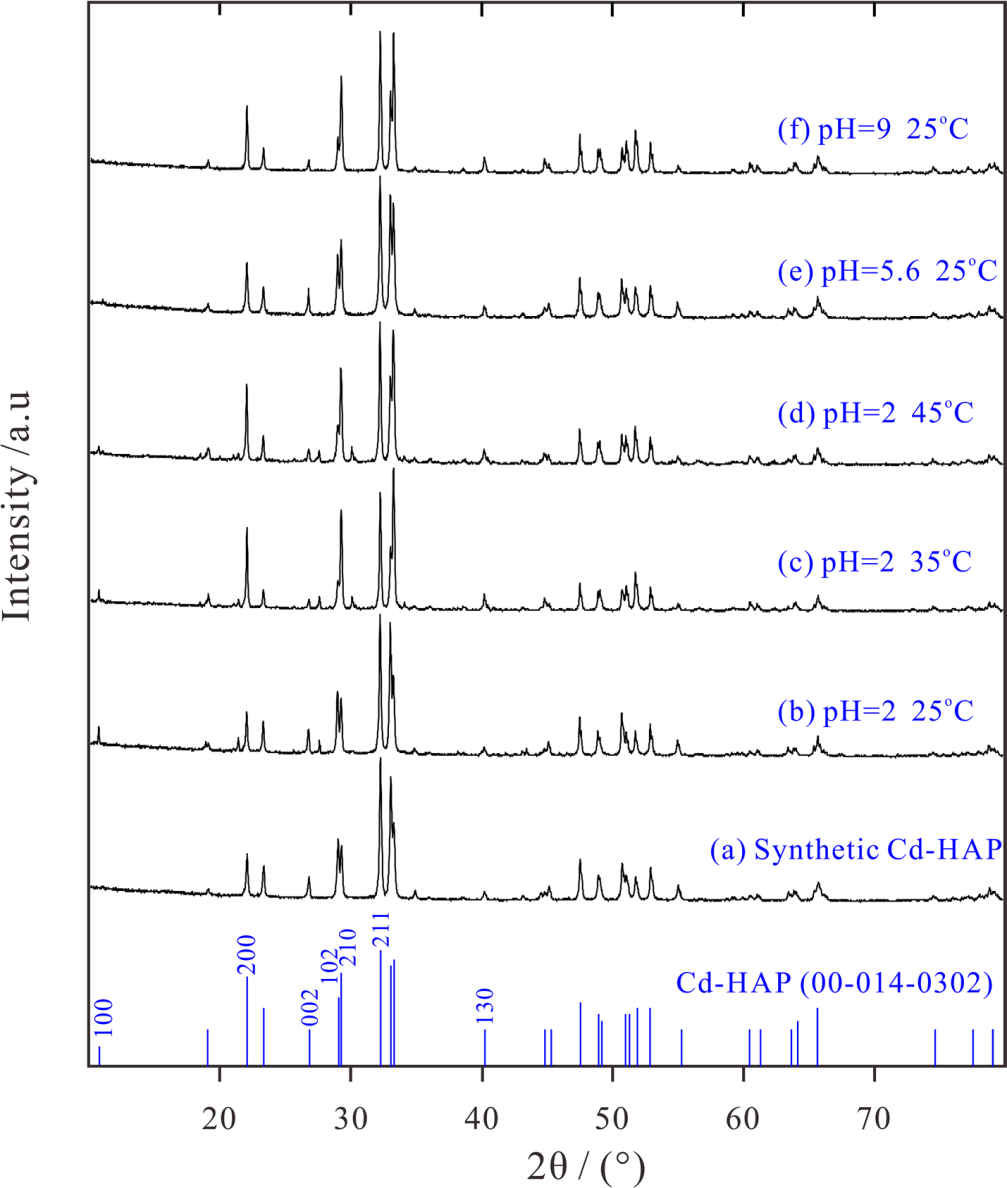
X-ray diffraction (XRD) patterns of the synthetic Cd-hydroxyapatite [Cd_5_(PO_4_)_3_OH] before (*a*) and after (*b*–*f*) dissolution at 25–45°C for 300 days.

**Figure 2:**
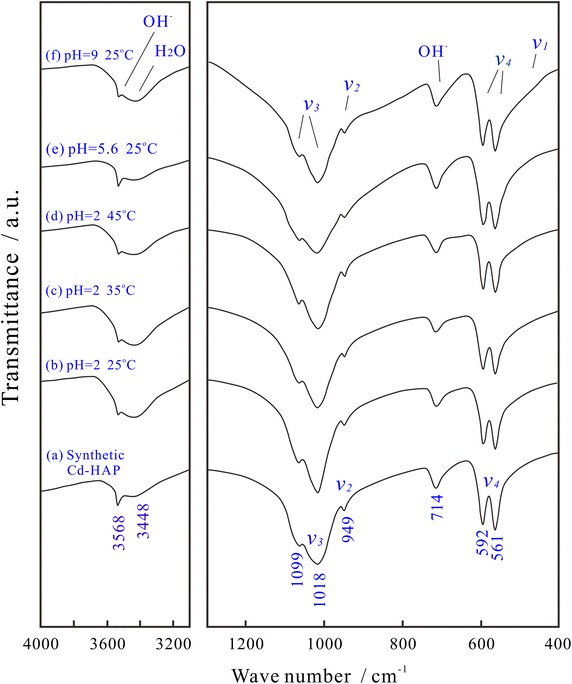
Fourier transform infrared (FT-IR) spectra of the synthetic Cd-hydroxyapatite [Cd_5_(PO_4_)_3_OH] before (*a*) and after (*b*–*f*) dissolution at 25–45°C for 300 days.

**Figure 3:**
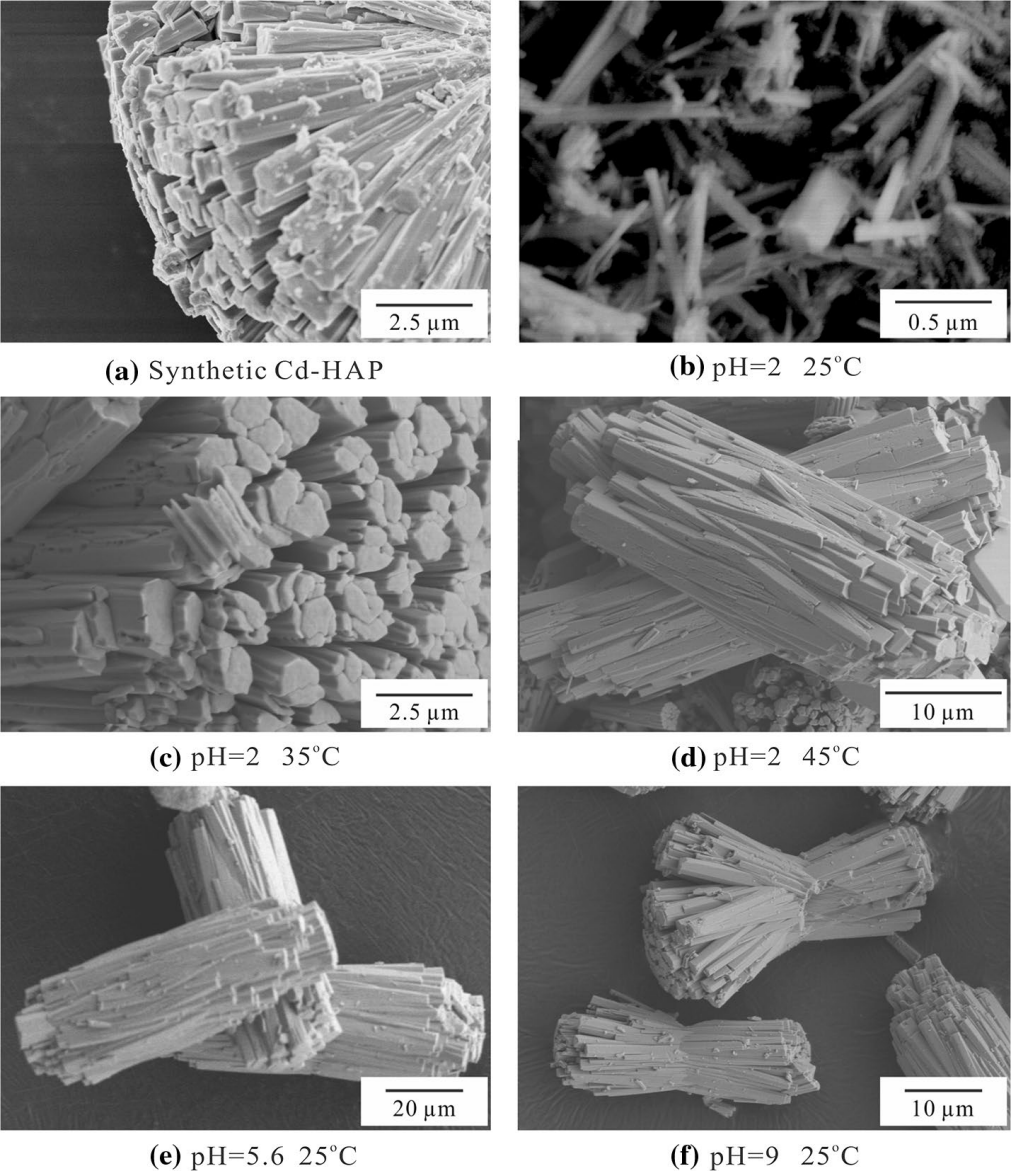
Field emission scanning electron microscopy (FE-SEM) images‎ of the synthetic Cd-hydroxyapatite [Cd_5_(PO_4_)_3_OH] before (**a**) and after (**b**–**f**) dissolution at 25–45°C for 300 days.

As showed in the XRD, FT-IR and FE-SEM figures, the character and component of the Cd-HAP solids were indistinguishable before and after the dissolution (Figures 1, 2 and 3). No secondary minerals were detected in the dissolution. The XRD patterns of the prepared solids indicated that all the products were identified as Cd-HAP (JCPDS Reference code 00-014-0302), which were highly crystallized and had the apatitic structure of the hexagonal system P6_3_/m with the estimated lattice parameters of *a* = 0.933 nm and *c* = 0.667 nm (Figure 1).

In the FT-IR spectra of the prepared solids (Figure 2), the normal vibrational modes of phosphate tetrahedra of cadmium hydroxylapatite (Cd-HAP) were observed within the region around at 949 cm^−1^ (*ν*_2_), 1,018 and 1,099 cm^−1^ (*ν*_3_), 561 and 592 cm^−1^ (*ν*_4_). The area of the phosphate peak at 474 cm^−1^ (*ν*_1_) was not visible. The spectrum of all samples possessed the very sharp bands at 3,568–3,570 cm^−1^ for the stretching vibrations of the bulk OH^−^. The 715 cm^−1^ bands were considered to be the librational motion of OH^−^ [5, 6]. The band at 871 cm^−1^, which was assigned to HPO_4_^2−^ ions contained in cation-deficient hydroxylapatite was not observed in our research [5]. The bands at 3,650–3,680 cm^−1^ for the surface P–OH groups [5] and the bands at 1,455 cm^−1^ for the CO_3_^2−^ vibration [20] were also not detected in the spectrum.

The FE-SEM results (Figure 3) showed that the Cd-HAP solids were the typical hexagonal columnar crystals with a pinacoid as a termination, which elongated along c axis (particle size 0.5–5 µm).

### Dissolution mechanism

**Figure 4:**
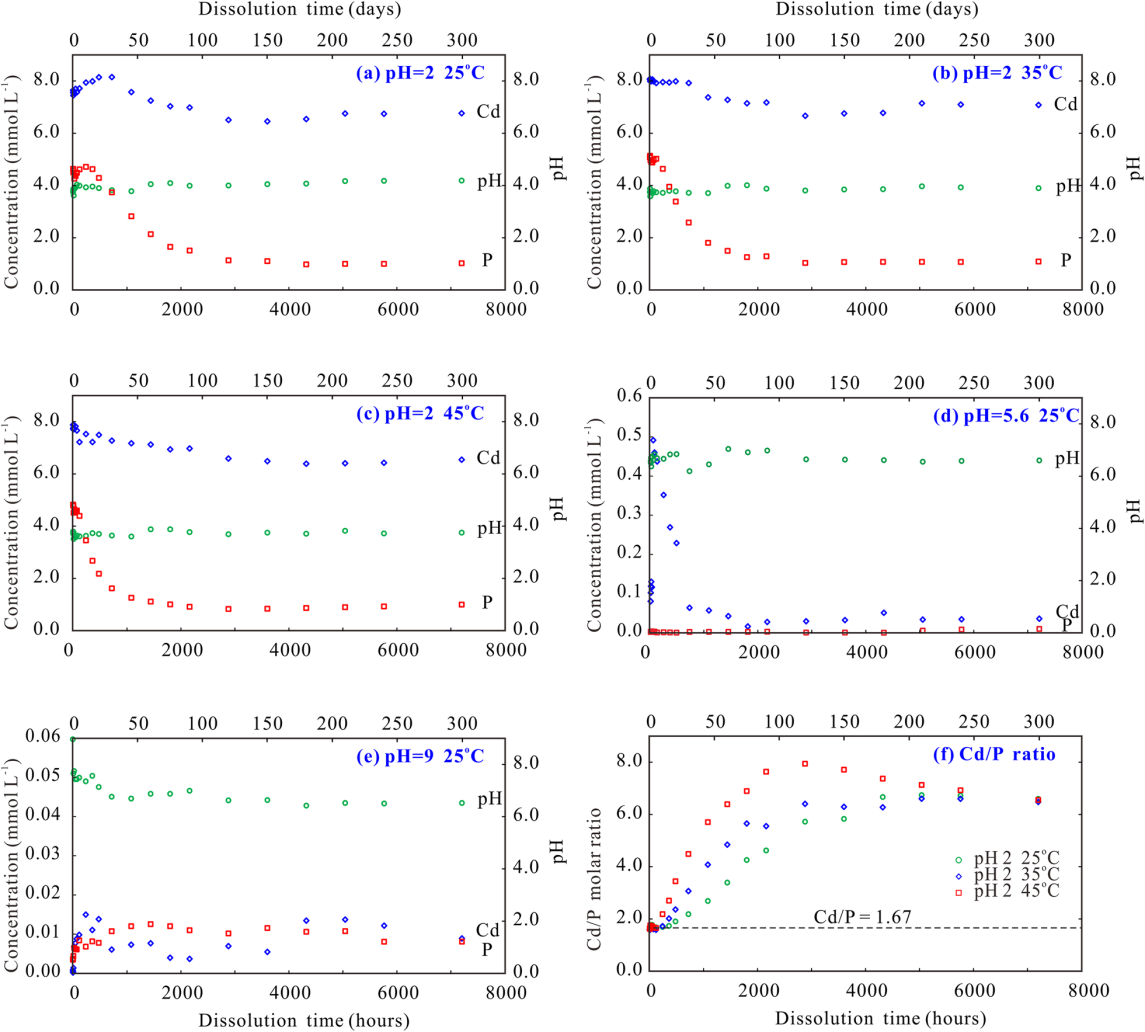
Aqueous evolution during the dissolution of the synthetic Cd-hydroxyapatite [Cd_5_(PO_4_)_3_OH] for 300 days. **a** Initial pH 2 and 25°C, **b** initial pH 2 and 35°C, **c** initial pH 2 and 45°C, **d** initial pH 5.6 and 25°C, **e** initial pH 9 and 25°C, and **f** the aqueous Cd/P molar ratios for the dissolution at initial pH 2 and 25°C, 35°C and 45°C, respectively.

The solution element concentrations and ratios for the Cd-HAP dissolution at 25, 35, 45°C and different pHs versus time are illustrated in Figure 4.

Dissolution of Cd-HAP in the acidic solution seemed to be stoichiometric in the early stage of dissolution and then always non-stoichiometric to the end of the dissolution experiments. For the dissolution of Cd-HAP at initial pH 2 and 25°C (Figure 4a), the cadmium and phosphate were rapidly released and the peak solution concentrations were reached after 20–30 days and 10 days for cadmium and phosphate, respectively. Thereafter, the Cd-HAP solid dissolved slowly while the solution Cd/P molar ratio increased constantly from 1.65 to 1.69 near the stoichiometric ratio of 1.67 for Cd-HAP, to 6.61–6.76 (300 days). The aqueous pH increased from 2.00 to 3.82 within the first hour of the dissolution and then fluctuated between 3.78 and 4.19. Besides, the solution temperature could influence the solution concentrations of cadmium and phosphate released from the Cd-HAP solid. At the end of the experiment, the aqueous cadmium and phosphate concentrations at 45°C were lower than those at 25°C. The transient peak value of the aqueous elemental concentration during the apatite dissolution has also been reported by earlier researchers [14], which was possibly owing to a particle size distribution. The smaller particles can raise a larger dissolution rate and a greater solubility. Therefore, the peak value showed in the figure may be a consequence of the fast dissolution of the finest particles with a greater solubility and the following re-precipitation to form bigger particles, which can result in the achievement of the asymptotic solubility valve at larger times [14].

In the early stage of the Cd-HAP dissolution (<5 days), cadmium and phosphate were released from the Cd-HAP solid surface to the aqueous solution at the stoichiometric Cd/P ratio of 1.67 (Figure 4f). With the increasing of the dissolution time, the solution Cd/P molar ratio increased and became greater than 1.67, which indicated that the Cd ions were preferentially released from the Cd-HAP surface in comparison with PO_4_^3−^. The solution Cd/P molar ratios at 45°C were significantly higher than those at 25°C, which indicated that the system temperature could obviously affect the dissolution process and solubility of Cd-HAP in aqueous solution. At the end of our experiment (300 days), the Cd/P molar ratios were near 6.50–6.61 for the Cd-HAP dissolution at 25–45°C and initial pH 2, i.e., there was no obvious variation of the Cd/P molar ratios with the solution temperature at equilibrium.

For the Cd-HAP dissolution in ultrapure water (pH = 5.60), after dissolution for 75 days, the solution pH, cadmium and phosphate concentrations appeared stable indicating the achievement of a steady-state between the solid and the solution (Figure 4d). With the initial release of Cd^2+^ and PO_4_^3−^ was the fast increasing of the aqueous pH from 5.60 to 6.58 in the first hour of dissolution, and after that the aqueous pH reached a stable state with the values of about 5.50–6.60 after dissolution for 120 days.

The H^+^ depleting indicated that the sorption of H^+^ onto negatively charged oxygen ions of phosphate groups of Cd-HAP can cause the transformation of surface PO_4_^3−^ into HPO_4_^2−^ groups and catalyze the reaction. Besides, the co-existence of exchange and dissolution reactions suggests that the H^+^ ions consumed during the Cd-HAP dissolution did not come only from sorption/desorption of H^+^ ions but from many different processes at the Cd-HAP surface. Therefore, a complete description of the H^+^ ions consumed during the dissolution should take account of the following processes: stoichiometric dissolution of the bulk Cd-HAP solid, stoichiometric exchange of 2H^+^ for one Cd^2+^ at the Cd-HAP surface, H^+^ adsorption/desorption at the Cd-HAP surface [15, 21]. The dissolution process of apatite is extremely related to the experimental conditions, e.g., temperature, agitation, solid component, solution composition, solid/solution ratio [15]. Different dissolution models of Ca-HAP have been suggested based on the previously published experimental data, all of them consider only some specific aspects of apatite dissolution and cannot express the dissolution process universally [15].

Based on the experimental results of the present work and some previous researches [15], the cadmium hydroxylapatite (Cd-HAP) dissolution in aqueous media is considered to have the following coincident steps or processes.

1. Stoichiometric dissolution coupled with protonation and complexation reactions.
2. Non-stoichiometric dissolution with Cd^2+^ release and PO_4_^3−^ sorption.
3. Sorption of Cd^2+^ and PO_4_^3−^ species from solution backwards onto Cd-HAP surface.
4. Steady state.

For the Cd-HAP dissolution in aqueous acidic media at initial pH 2 and 25°C, in the Processes 1 and 2, Cd^2+^ and PO_4_^3−^ in the Cd-HAP structure can be removed coincidentally/stoichiometrically with the dissolution rates in proportion to the molar concentrations according to the reaction (1), i.e., the aqueous cadmium and phosphate concentration decreased with time simultaneously at the aqueous Cd/P molar ratio of 1.67 in the early period of dissolution (0–10 days).

Many possible reactions should be considered in the research on the apatite dissolution due to its structural complexity [3]. The reaction (1) for the Cd-HAP dissolution is strongly influenced by the solution pH and combined with the protonation and complexation reactions (2) and (3), which resulted in the aqueous pH increasing for the dissolution in aqueous acidic media or the aqueous pH decreasing for the dissolution in aqueous alkali media.

1 $${\text{Cd}}_{ 5} \left( {{\text{PO}}_{ 4} } \right)_{ 3} {\text{OH}}{ \leftrightharpoons } 5 {\text{ Cd}}^{ 2+ } + 3 {\text{ PO}}_{ 4}^{ 3- } + {\text{OH}}^{ - }$$

2 $${\text{PO}}_{ 4}^{ 3- } + {\text{nH}}^{ + } { \leftrightharpoons }{\text{H}}_{\text{n}} {\text{PO}}_{ 4}^{{\left( { 3- {\text{n}}} \right) - }} \quad \left( {{\text{n }} = { 1},{ 2},{ 3}} \right)$$

3 $${\text{Cd}}^{ 2+ } + {\text{nOH}}^{ - } { \leftrightharpoons }{\text{Cd}}\left( {\text{OH}} \right)_{\text{n}}^{{({\text{n}} - 2) - }} \quad \left( {{\text{n }} = { 1},{ 2},{ 3},{ 4}} \right)$$

The speciation results based on the calculation with PHREEQC indicated that for the Cd-HAP dissolution at 25°C and initial pH 2, the aqueous cadmium species existed in the order of Cd^2+^ > CdOH^+^ > Cd_2_OH^3+^ > Cd(OH)_2_ > Cd(OH)_3_^−^ > Cd(OH)_4_^2−^; the aqueous phosphate species occurred in the order of H_2_PO_4_^−^ > H_3_PO_4_ > HPO_4_^2−^ > PO_4_^3−^. At the early stage of dissolution, Cd^2+^ and Cd_2_OH^3+^ increased with time, CdOH^+^ is stable, Cd(OH)_2_, Cd(OH)_3_^−^ and Cd(OH)_4_^2−^ decreased with time. H_2_PO_4_^−^ and H_3_PO_4_ increased with time, HPO_4_^2−^ is stable, PO_4_^3−^ decreased with time. For the Cd-HAP dissolution at 25°C and initial pH 9, the aqueous cadmium species existed in the order of Cd^2+^, CdOH^+^ > Cd(OH)_2_ > Cd_2_OH^3+^ > Cd(OH)_3_^−^ > Cd(OH)_4_^2−^; the aqueous phosphate species occurred in the order of HPO_4_^2−^ > H_2_PO_4_^−^ > PO_4_^3−^ > H_3_PO_4_ as the dissolution time <20 days or in the order of H_2_PO_4_^−^ > HPO_4_^2−^ > H_3_PO_4_ > PO_4_^3−^ as the dissolution time >20 days.

In processes 2, cadmium and phosphate are removed non-coincidentally/non-stoichiometrically from the Cd-HAP structure with different dissolution rates and the aqueous Cd/P molar ratio >1.67. As a result, a surface layer, which has a chemical component different from that of the bulk solid, can be formed [15]. When an initial portion of Cd-HAP had been dissolved, some amount of phosphate could be adsorbed from the solution backwards onto the Cd-HAP surface. In this work, the solution phosphate concentrations began to decline gradually after 10 days dissolution at initial pH 2 and 25°C, whereas the solution cadmium concentration increased constantly with time. The aqueous Cd/P molar ratio increased from 1.72 to about 3.07 in the dissolution between 10 and 30 days.

In processes 3, both of cadmium and phosphate ions were adsorbed simultaneously from the aqueous solution backwards onto the Cd-HAP surface, the aqueous cadmium and phosphate ions concentration decreased from 30 to 210 days with the increasing aqueous Cd/P molar ratio (3.07–6.69), which can result in the forming of a surface layer having a component different from that of the bulk of Cd-HAP [15], i.e., the component of the final equilibrated solution could be dependent on a surface layer with a component different from that of Cd-HAP [Cd_5_(PO_4_)_3_(OH)]. The Cd/P ratios were found to be 6.50–6.61 for the Cd-HAP dissolution at 25–45°C and initial pH 2. For the Ca-HAP dissolution in 0.1 M NaCl at near neutral pH values and 25°C, the solution Ca/P molar ratio was observed to have values even as great as 25 [3]. Due to its low solubility, the apatite dissolution mechanism may be dependent on several chemical reactions happening simultaneously at the apatite surface [22], and it is considered that the apatite dissolution is always non-stoichiometric at the atomic level [15].

In processes 4, desorption and adsorption of cadmium and phosphate ions reached a steady state, i.e., the aqueous cadmium and phosphate ions concentration were stable for the Cd-HAP dissolution in aqueous acidic media at initial pH 2 and 25°C from 210 days to the experimental end (300 days), and the aqueous Cd/P molar ratios remained stable (6.61–6.76).

### Determination of solubility

The activities of the aqueous cadmium and phosphate species present in the final equilibrated solutions (210, 240, 300 days) were used to determine the solubility product for cadmium hydroxylapatite [Cd_5_(PO_4_)_3_OH]. The aqueous solutions were calculated to be undersaturated with respect to any possible secondary minerals (e.g. Cd_3_(PO_4_)_2_, Cd(OH)_2_(am), Cd(OH)_2_, monteponite).

The dissolution of cadmium hydroxylapatite and the release of cadmium and phosphate can be described by the reaction Eq. (1). Supposing unit activity of the solid phase

4 $$K_{\text{sp}} = \, \left\{ {{\text{Cd}}^{ 2+ } } \right\}^{ 5} \left\{ {{\text{PO}}_{ 4}^{ 3- } } \right\}^{ 3} \left\{ {{\text{OH}}^{ - } } \right\}$$

where {} represents the thermodynamic activities of the aqueous species and *K*_sp_ is the equilibrium constant of the reaction Eq. (1).

The standard free energy of reaction ($\Delta G_{r}^{o}$), in kJ/mol, is dependent on *K*_sp_ under the standard condition (298.15 K and 0.101 MPa) by

5 $$\Delta G_{r}^{o} = - 5. 70 8 {\text{ log}}K_{\text{sp}}$$

For Eq. (1),

6 $$\Delta G_{r}^{o} = 5\Delta G_{f}^{o} \left[ {{\text{Cd}}^{ 2+ } } \right] + 3\Delta G_{f}^{o} \left[ {{\text{PO}}_{ 4}^{ 3- } } \right] + \Delta G_{f}^{o} [{\text{OH}}^{ - } ] - \Delta G_{f}^{o} \left[ {{\text{Cd}}_{ 5} \left( {{\text{PO}}_{ 4} } \right)_{ 3} {\text{OH}}} \right]$$

Rearranging,

7 $$\Delta G_{f}^{o} \left[ {{\text{Cd}}_{ 5} \left( {{\text{PO}}_{ 4} } \right)_{ 3} {\text{OH}}} \right] = 5\Delta G_{f}^{o} \left[ {{\text{Cd}}^{ 2+ } } \right] + 3\Delta G_{f}^{o} \left[ {{\text{PO}}_{ 4}^{ 3- } } \right] + \Delta G_{f}^{o} [{\text{OH}}^{ - } ] - \Delta G_{r}^{o}$$

Table 1 lists the pH, Cd and P analyses at different initial solution pHs and temperatures, as well as the calculated solubility products (*K*_sp_) for Cd-HAP. The aqueous activities of Cd^2+^(aq), PO_4_^3−^(aq) and OH^−^(aq) were first calculated via the computer program PHREEQC [19], and then the *K*_sp_ values for Cd-HAP [Cd_5_(PO_4_)_3_OH] were calculated according to Eq. (4). The mean *K*_sp_ values were determined for Cd-HAP [Cd_5_(PO_4_)_3_OH] of 10^−64.62^ (10^−64.53^–10^−64.71^) at 25°C, 10^−65.58^ (10^−65.31^–10^−65.80^) at 35°C and 10^−66.57^ (10^−66.24^–10^−66.90^) at 45°C.

**Table 1:** Analytical data and solubility determination of cadmium hydroxylapatite [Cd_5_(PO_4_)_3_OH]

Temp (^o^C)	Initial pH	Dissolution time (days)	Analytical data			log*K*_sp_	$\Delta G_{f}^{o}$ (kJ/mol)
			pH	Cd (mmol/L)	P (mmol/L)		
25	2	210	4.17	6.7556	0.9995	−64.71	−3,970.96
		240	4.18	6.7457	1.0008	−64.63	−3,970.53
		300	4.19	6.7631	1.0225	−64.53	−3,969.92
35	2	210	3.97	7.1479	1.0797	−65.31	
		240	3.93	7.0999	1.0732	−65.61	
		300	3.90	7.0839	1.0906	−65.80	
45	2	210	3.82	6.4051	0.8956	−66.24	
		240	3.72	6.4269	0.9253	−66.90	
		300	3.75	6.5443	0.9957	−66.56	
25	5.6	210	6.55	0.0337	0.0054	−58.59	−4,174.00
		240	6.58	0.0343	0.0083	−58.96	−4,176.11
		300	6.60	0.0358	0.0097	−59.41	−4,178.69
25	9	210	6.52	0.0137	0.0108	−66.71	−3,982.36
		240	6.50	0.0121	0.0080	−67.47	−3,986.74
		300	6.52	0.0089	0.0081	−68.01	−3,989.80

Based on the obtained solubility data from the dissolution at initial pH 2 and 25°C, the Gibbs free energies of formation $\Delta G_{f}^{o}$[Cd_5_(PO_4_)_3_OH] were calculated using Eqs. (5), (6), and (7) to be −3,970.47 kJ/mol (−3,969.92 to −3,970.96 kJ/mol).

No reports on the solubility properties of Cd-HAP at 25°C have appeared in the previous studies. The mean *K*_sp_ values 10^−64.62^ (10^−64.53^–10^−64.71^) for Cd_5_(PO_4_)_3_OH at 25°C, 10^−65.58^ (10^−65.31^–10^−65.80^) at 35°C and 10^−66.57^ (10^−66.24^–10^−66.90^) at 45°C were approximately 9.40–11.35 log units lower than 10^−55.22^ reported for Cd_5_(PO_4_)_3_OH at 37°C [17], which is the only solubility data found until now, but only one data point was used to calculate the solubility product for Cd-HAP. Besides, their solubility value was determined in aqueous solution at pH 5.175 and 37°C under a fixed ionic environment using 0.165 M NaNO_3_, and the solid was thought to have achieved equilibrium, but the authors themselves indicated that the dicadmium phosphate (CdHPO), not the Cd-HAP, seemed to control the solubility equilibrium at pH 5.0–7.0.

The mean *K*_sp_ value was determined for Ca-HAP [Ca_5_(PO_4_)_3_OH] of 10^−57.65^ (10^−57.56^–10^−57.79^) at 25°C in this study. The thermodynamic solubility products (*K*_sp_) for Ca-HAP [Ca_5_(PO_4_)_3_OH] were reported to be 10^−58.3^ [23], 10^−57^ [24], 10^−59^ [25], 10^−58±1^ [13], and 10^−57.72^ [26]. In comparison with the solubility products of Ca-HAP reported in literature, the mean *K*_sp_ value 10^−64.62^ (10^−64.53^–10^−64.71^) for Cd-HAP was approximately 7.62–5.62 log units lower than 10^−57^–10^−59^ for Ca-HAP. A *K*_sp_ value for lead hydroxylapatite (Pb-HAP) has been reported to be approximately 10^−76.71^ [27], while a *K*_sp_ value of 10^−62.79^ was given for Pb-HAP in the minteq.v4.dat database [19]. Comparison of the solubility products (*K*_sp_) of Ca-HAP and lead hydroxylapatite (Pb-HAP) indicates that the conversion of Ca-HAP to Pb-HAP is thermodynamically favorable in the presence of solution Pb^2+^ [28]. In harmony with Pb-HAP, the solubility product of Cd-HAP is extremely low, i.e., several orders of magnitude less soluble than Ca-HAP.

### Determination of thermodynamic data

The solubility products (*K*_sp_) for Cd-HAP and Ca-HAP can be expressed through Eqs. (8)–(10) [17].

8 $$- { \log }K_{\text{sp}} = A/{\text{T}} + B + C{\text{T}}$$

where *A*, *B* and *C* are the empirical constants of Eq. (8).

For Cd-HAP

9 $$- { \log }K_{\text{sp}} = 5 2 3 9. 1 2 3/{\text{T}} + 1. 6 2 6 9 9+ 0. 1 5 2 4 {\text{ T}}$$

For Ca-HAP

10 $$- { \log }K_{\text{sp}} = 3 9 , 5 2 4. 4 8 5/{\text{T}} - 2 2 3. 4 9 7 5 1+ 0. 4 9 8 3 6\;{\text{T}}$$

The thermodynamic parameters Δ*G*^0^, Δ*H*^0^, Δ*S*^0^ and $\Delta C_{\text{p}}^{0}$ for the dissolution of Cd-HAP and Ca-HAP at initial pH 2.0 listed in Table 2 are obtained from Eqs. (11)–(14) [17].

**Table 2:** Thermodynamic data of Cd-HAP and Ca-HAP in aqueous acidic media (initial pH 2)

HAP	Temp (^o^C)	Δ*G* ^0^ (J/K mol)	Δ*H* ^0^ (J/mol)	Δ*S* ^0^ (J/K mol)	$\Delta C_{\text{p}}^{0}$ (J/K mol)
Ca-HAP	25	328,903.30	−90,597.07	−1,407.72	−2,843.41
	35	343,934.66	−148,419.53	−1,598.55	−2,938.83
	45	360,874.35	−208,150.32	−1,789.39	−3,034.25
Cd-HAP	25	368,710.12	−158,809.53	−1,770.20	−869.52
	35	386,703.91	−176,491.82	−1,828.56	−898.70
	45	405,281.27	−194,757.67	−1,886.91	−927.88

11 $$\Delta G^{ 0} = 2. 30 2 6\;R\left( {A + B{\text{T}} + C{\text{T}}^{ 2} } \right)$$

12 $$\Delta H^{ 0} = 2. 30 2 6\;R\left( {A - C{\text{T}}^{ 2} } \right)$$

13 $$\Delta S^{ 0} = - 2. 30 2 6\;R\left( {B + 2C{\text{T}}} \right)$$

14 $$\Delta C_{\text{p}}^{0} = - 2. 30 2 6\;R\left( {C{\text{T}}} \right)$$

The negative Δ*H*^0^ values suggested that the Cd-HAP and Ca-HAP dissolution in aqueous solution was an exothermic process and their solubilities decreased with increasing temperature. The larger negative Δ*S*^0^ values for the Cd-HAP dissolution showed that the order caused by Cd^2+^ in aqueous solution was higher than that produced by Ca^2+^ [17], which was considered to be related to the different sizes of Cd^2+^ (0.97 Å) and Ca^2+^ (0.99 Å). The smaller Cd^2+^ with a high charge can cause larger entropy in aqueous media, and therefore Cd-HAP is less soluble than Ca-HAP. The Δ*G*^0^, Δ*H*^0^ and Δ*S*^0^ values were greater for Cd-HAP than Ca-HAP in solution, suggesting that the Cd-HAP dissolution was more energy consuming and consequently it is less soluble [17].

## Conclusions

The XRD pattern showed that the prepared solids were pure cadmium hydroxylapatite (Cd-HAP) with the estimated lattice parameters of *a* = 0.933 nm and *c* = 0.667 nm. In FT-IR spectra, the normal vibrational modes of phosphate tetrahedra for Cd-HAP appeared around 949 cm^−1^ (*ν*_2_), 1,018 and 1,099 cm^−1^ (*ν*_3_), 561 and 592 cm^−1^ (*ν*_4_). The FE-SEM observation indicated the solid was the typical hexagonal columnar crystals with a pinacoid as a termination that elongated along the *c* axis (particle sizes 0.5–5 µm). Characterization by XRD, FT-IR and FE-SEM proved that Cd-HAP solids showed no recognizable change during dissolution.

For the Cd-HAP dissolution in aqueous acidic media at initial pH 2 and 25°C, the solution cadmium and phosphate concentrations increased rapidly and reached the peak values after 20–30 days and 10 days reaction, respectively. Thereafter, the Cd-HAP dissolution rate decreased slowly, whereas the solution Cd/P molar ratio increased constantly from 1.65–1.69 to 6.61–6.76. The mean *K*_sp_ values for Cd_5_(PO_4_)_3_OH were determined to be 10^−64.62^ (10^−64.53^–10^−64.71^) at 25°C, 10^−65.58^ (10^−65.31^–10^−65.80^) at 35°C and 10^−66.57^ (10^−66.24^–10^−66.90^) at 45°C. Based on the obtained solubility data from the dissolution at initial pH 2 and 25°C, the Gibbs free energy of Cd_5_(PO_4_)_3_OH forming $\Delta G_{f}^{o}$ was determined to be −3,970.47 kJ/mol (−3,969.92 to −3,970.96 kJ/mol). Thermodynamic quantities, Δ*G*^0^, Δ*H*^0^, Δ*S*^0^, and $\Delta C_{\text{p}}^{0}$ for the dissolution process of Cd-HAP in aqueous acidic media at initial pH 2 and 25°C were calculated 368,710.12 J/K mol, −158,809.54 J/mol, −1,770.20 J/K mol and −869.53 J/K mol, respectively.
